# Highly efficient correction of structural mutations of 450 kb *KIT* locus in kidney cells of Yorkshire pig by CRISPR/Cas9

**DOI:** 10.1186/s12860-019-0184-5

**Published:** 2019-04-03

**Authors:** Ke Qin, Xinyu Liang, Guanjie Sun, Xuan Shi, Min Wang, Hongbo Liu, Yaosheng Chen, Xiaohong Liu, Zuyong He

**Affiliations:** State Key Laboratory of Biocontrol, School of Life Sciences, Sun Yat-sen University, Guangzhou, 510006 People’s Republic of China

**Keywords:** CRISPR/Cas9, *KIT*, Pig, Structural variation

## Abstract

The white coat colour of Yorkshire and Landrace pig breeds is caused by the dominant white *I* allele of *KIT*, associated with 450-kb duplications and a splice mutation (G > A) at the first base in intron 17. To test whether genome editing can be employed to correct this structural mutation, and to investigate the role of *KIT* in the control of porcine coat colour, we designed sgRNAs targeting either intron 16 or intron 17 of *KIT*, and transfected Cas9/sgRNA co-expression plasmids into the kidney cells of Yorkshire pigs. The copy number of *KIT* was reduced by about 13%, suggesting the possibility of obtaining cells with corrected structural mutations of the *KIT* locus. Using single cell cloning, from 24 successfully expanded single cell clones derived from cells transfected with sgRNA targeting at intron 17, we obtained 3 clones with a single copy of *KIT* without the splice mutation. Taken together, the 12.5% (3/24) efficiency of correction of structural mutations of 450 kb fragments is highly efficient, providing a solid basis for the generation of genome edited Yorkshire pigs with a normal *KIT* locus. This provides an insight into the underlying genetic mechanisms of porcine coat colour.

**Electronic supplementary material:**

The online version of this article (10.1186/s12860-019-0184-5) contains supplementary material, which is available to authorized users.

## Background

Artificial selection in different regions of the world has strongly accelerated porcine evolution and has resulted in pig coat colour variations in contrast to their wild ancestors [5]. Variability in several genes has been shown to affect pigmentation in pigs. Among them, *KIT* (Dominant White locus) may play a major role in determining the white coat colour in the Yorkshire and Landrace pig breeds. *KIT* was previously mapped to chromosome 8 of pigs, encoding the proto-oncogene receptor tyrosine kinase, which plays a crucial role in the survival and migration of neural-crest–derived melanocyte precursors [2]. Four alleles have been identified at the dominant white locus: the recessive *i* allele for wild-type solid colour, the semi-dominant *I*^*p*^ allele for the patch phenotype, the fully dominant *I* allele for the dominant white phenotype, and *I*^*Be*^ for the dominant belt phenotype [16]. A splice mutation (G > A) at the first base of intron 17 in a 450 kb duplication is found in *I* allele of Yorkshire and Landrace pigs, which is assumed to act as a regulatory mutation and has a phenotypic effect due to the overexpression or dysregulated expression of *KIT* [10, 14]. However, this assumption has not yet been validated by functional studies, mainly due to the difficulty associated with correcting structural mutations of a 450-kb locus. The emergence of genome editing technology may provide us with an opportunity to overcome this problem. The clustered regularly interspaced short palindromic repeats (CRISPR) and CRISPR-associated (Cas) system has become a powerful and versatile tool for genome engineering. The CRISPR/Cas9 system is composed of two components: a single guide RNA (sgRNA) and a Cas9 endonuclease. The sgRNA is composed of a target-specific CRISPR RNA (crRNA) and an auxiliary trans-activating crRNA (tracrRNA). It can guide the Cas9 protein to a specific genomic locus via base pairing between the crRNA sequence and the target sequence [3, 9]. Subsequently, Cas9 produces site-specific DNA double-strand breaks (DSBs), which can stimulate DNA repair pathways via two competitive mechanisms, homologous recombination (HR) or non-homologous end-joining (NHEJ), where the NHEJ process is dominant and prone to generate targeted mutagenesis [21]. In recent years, the CRISPR/Cas9 system has been widely employed in genome editing, including endogenous gene disruption, targeted sites insertion, and chromosomal rearrangements, in various organisms ranging from viruses to eukaryotes since its development [11, 19, 20], with advantages including easy programmability, wide applicability, and time saving.

Here, we employed CRISPR/Cas9 to delete the duplicated copies of the 450-kb *KIT* locus and eliminate the splice mutation in kidney cells of Yorkshire pigs. The aim was to obtain donor cells with a normal *KIT* locus for somatic nuclear transfer in order to generate genome-edited Yorkshire pigs for further investigation ofthe molecular control mechanisms of *KIT* on the coat colour of pigs, and provided an insight into the generation of a new breed of Yorkshire pigs with wild-type coat colour.

## Results

### Efficient cutting at KIT locus in porcine kidney cells by CRISPR/Cas9

**Fig. 1:**
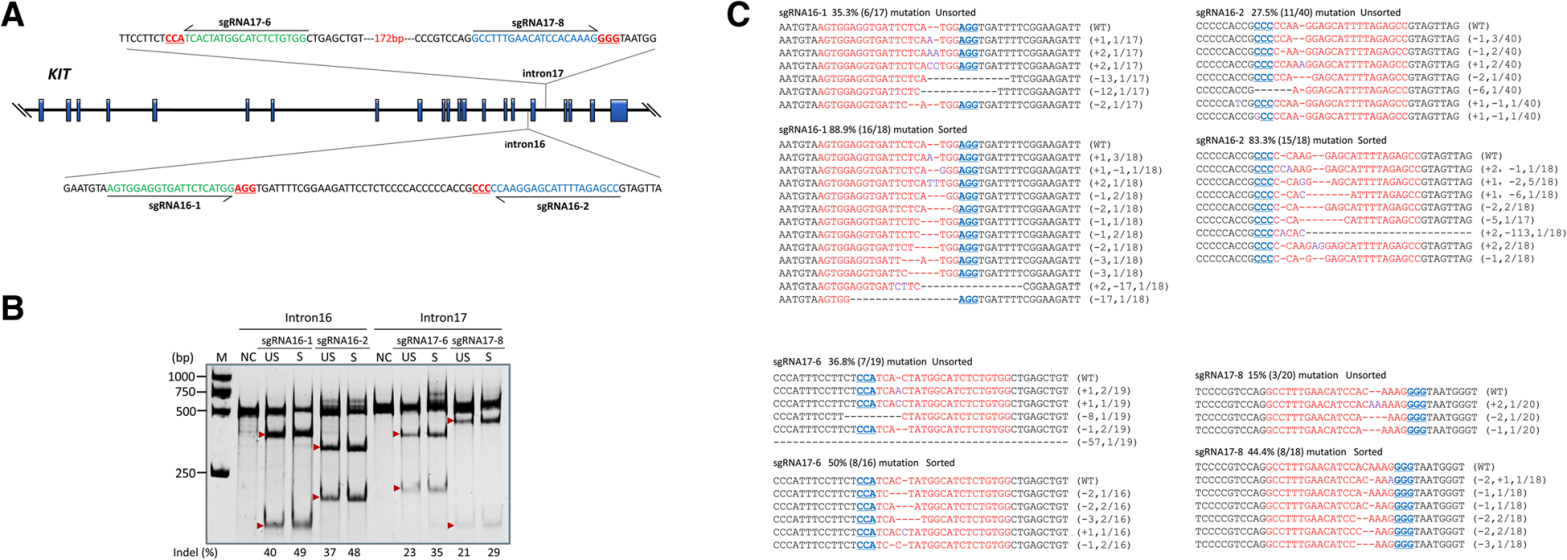
sgRNAs design and targeting efficiency measurement. **a** Schematic diagram of the target sites of sgRNAs designed for targeting introns 16 and 17 of the porcine *KIT* gene. Blue rectangles indicate exons and dark lines indicate introns. Half arrows indicate the sequence of the guide segment of sgRNAs. Red bases represent the NGG nucleotide protospacer adjacent motif (PAM) sequences. **b** The frequency of CRISPR/Cas9-induced mutations determined by the T7E1 assay. M, DNA marker; NC, negative control; US, unsorted; S, sorted. Red arrowheads indicate the expected positions of DNA bands cleaved by mismatch-sensitive T7E1. The numbers along the bottom of the gel indicate the mutation percentages calculated based on the band intensities using Image J software. **c** Sequence analysis of cloned PCR products. DNA sequences of the wild-type (WT) and mutant clones, with CRISPR/Cas9 recognition sites shown in red and PAM sequences in blue. Dashes and purple letters indicate deleted and inserted bases, respectively

To evaluate the targeting efficiencies of the designed sgRNAs (Fig. 1a and Additional file 1: Table S1) at the *KIT* locus in the kidney cells of Yorkshire pigs, firstly, genomic DNA of cells with four copies of the *KIT* locus transfected with pX458-sgRNAs (Additional file 2:Figure. S1) were subjected to digestion by hetero-duplex DNA sensitive T7E1. Significant cleavage bands at target T7E1 demonstrated that each sgRNA was able to efficiently induce NHEJ at its target site. The two sgRNAs targeting intron 16 presented a relative higher efficiency (40% for sgRNA16–1; 37% for sgRNA16–2) compared with the two sgRNAs targeting intron 17 (23% for sgRNA17–6; 21% for sgRNA17–8) (Fig. 1b). Transfection followed by sorting the EGFP positive cells by FACS (Additional file 3:Figure. S2) was found to effectively enrich cells transfected with sgRNA and thus improved the proportion of edited cells (Fig. 1b). The T7E1 assay tended to underestimate sgRNAs with higher mutation frequencies because mutant sequences can form homoduplexes, which are insensitive to T7E1 digestion [17]. Therefore, we further cloned the PCR amplicons containing the sgRNA target sites into the pMD18-T simple vector for Sanger sequencing to quantify the NHEJ events. In unsorted cells, the mutation frequencies induced by sgRNAs (35.3% for sgRNA16–1; 27.5% for sgRNA16–2; 36.8% for sgRNA17–6; 15.0% for sgRNA17–8) were close to those estimated by the T7E1 assay, while in sorted cells, the mutation frequencies (88.9% for sgRNA16–1; 83.3% for sgRNA16–2; 50.0% for sgRNA17–6; 44.4% for sgRNA17–8) induced by the sgRNAs were significantly underestimated by T7E1 assay (Fig. 1c).

### Copy number reduction detected in cell populations edited by CRISPR/Cas9

**Fig. 2:**
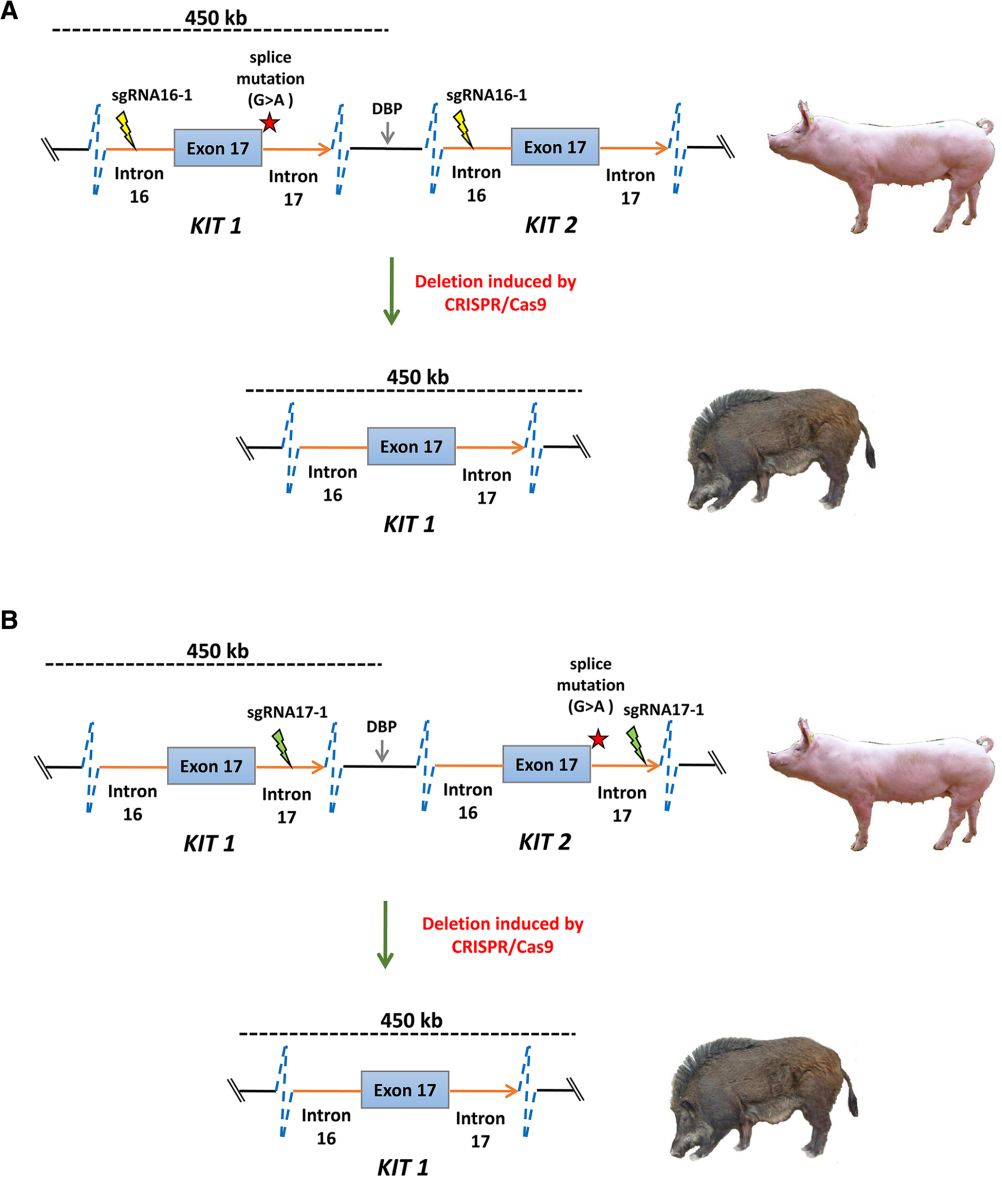
Removal of duplicated *KIT* copy by CRISPR/Cas9 from single allele with two *KIT* locus copies. **a** Schematic diagram of the strategy for removing the duplicated *KIT* copy when the KIT copy with the splice mutation is upstream of the normal KIT copy. **b** Schematic diagram of the strategy for removing the duplicated *KIT* copy when the KIT copy with the splice mutation is downstream of the normal KIT copy. Dashed line indicates that the length of the duplication region is 450 kb. DBP denotes the breakpoint of the duplication region. The red star indicates the splice mutation at the first nucleotide in intron 17 of the *KIT* gene

**Fig. 3:**
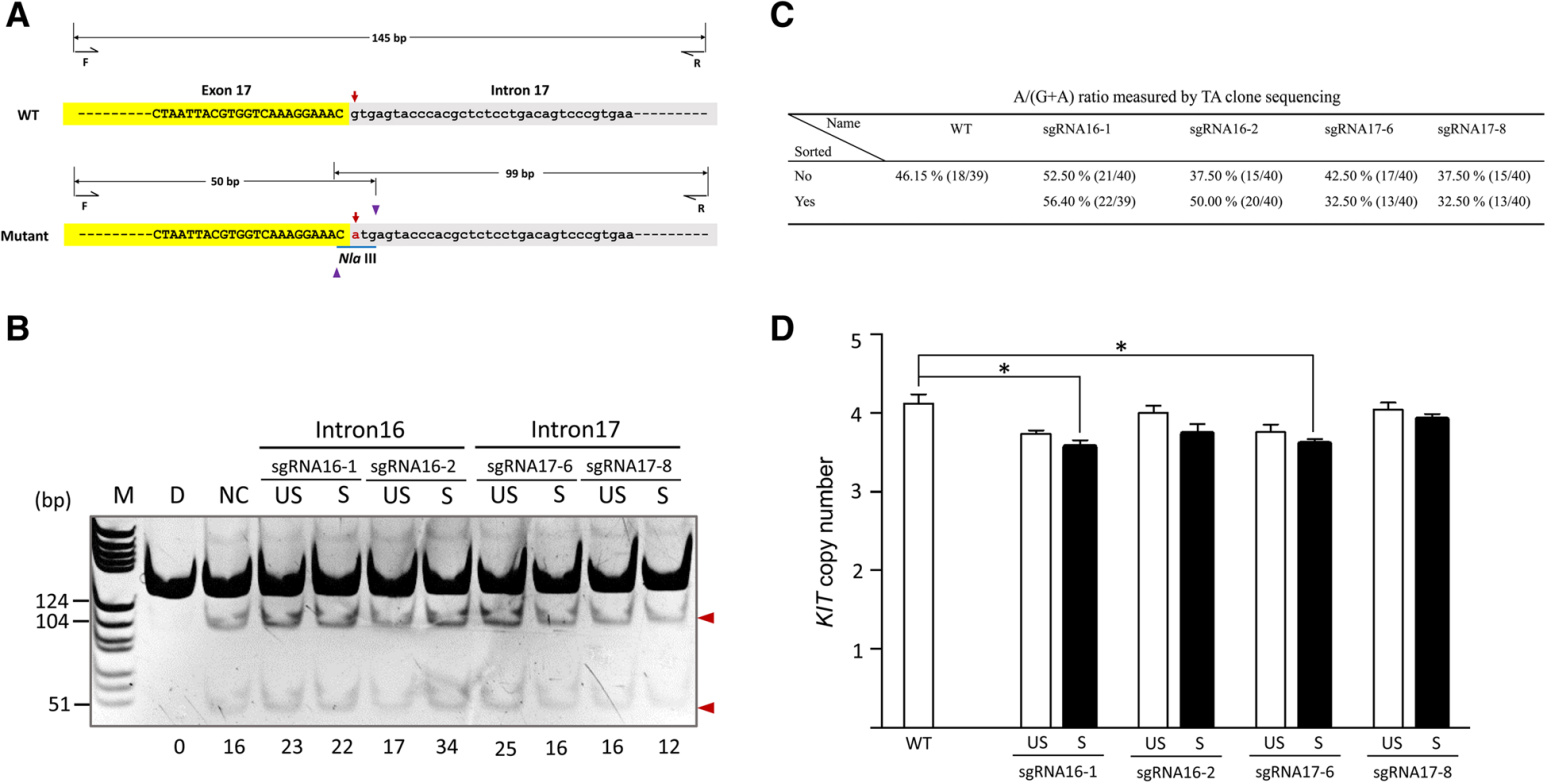
Measuring *KIT* copy number reduction in porcine kidney cells transfected with CRISPR/Cas9. **a** Schematic illustration of the *Nla* III assay. Red arrows indicate the first nucleotide in intron 17. The G > A mutation introduces an *Nla* III restriction site, as labelled by the blue underline. The bases with a yellow background represent exon 17 and those with a grey background represent intron 17. Blue arrowheads indicate the cutting sites of *Nla* III. **b** Variation of the percentage of *KIT* copy with the splice mutation in cell populations transfected with CRISPR/Cas9 as determined by the *Nla* III assay. M, DNA ladder; D, duroc pig (pig breed with wild-type *KIT* allele); NC, negative control, denotes the unedited Yorkshire pig kidney cells; US, unsorted; S, sorted. Red arrowheads indicate the expected positions of DNA bands cleaved by *Nla* III. The numbers along the bottom of the gel indicate the A/(G + A) ratio calculated based on the band intensities using Image J software. **c** The A/(G + A) ratio in cell populations transfected with CRISPR/Cas9 as measured by TA clone sequencing analysis. **d** *KIT* copy number variations in cell populations transfected with CRISPR/Cas9 as determined by qPCR (T-test, *p* < 0.05)

Since the designed sgRNAs were able to guide the Cas9 to cut at the target sites efficiently, we further investigated whether they were capable of deleting the duplicated copies of the 450-kb *KIT* locus with the splice mutation, thus correcting the structural mutation. The relative order of *KIT* copies (with and without the splice mutation) has not yet been established. However, it is possible to determine the order in the allele with only one duplicated copy by CRISPR/Cas9. If the copy with splice mutation is upstream of the normal copy, efficient deletion induced by sgRNA targeting intron 16 will remove the mutated copy, and the normal copy will remain in the genome (Fig. 2a). In contrast, if the mutated copy is downstream of the normal copy, efficient deletion induced by sgRNA targeting intron 17 will correct the structural mutation (Fig. 2b). Successful deletion of the *KIT* copy with the splice mutation will affect the G/A ratio in the first nucleotide of intron 17. This could be detected by *Nla* III assay (Fig. 3a). sgRNAs targeting intron 16 increased *Nla* III digestion, especially in cells sorted by FACS, which indicated *KIT* copies with splice mutation is downstream of the normal *KIT* copy (Fig. 3b). On the other hand, sgRNAs targeting intron 17 had no apparent effect on *Nla* III digestion (Fig. 3b), suggesting that *Nla* III digestion has limitation in detecting copy number variations in a small fraction of cells. Therefore, we further cloned the PCR amplicons containing the splice mutation into the pMD18-T simple vector for Sanger sequencing to quantify the G/A ratio. sgRNAs targeting intron 16 were clearly found to increase the percentage of splice mutations, especially in cells sorted by FACS, while sgRNAs targeting intron 17 reduced the percentage of splice mutations in both sorted and unsorted cells (Fig. 3c). This result further implies that the *KIT* copy with the splice mutation sites is downstream of the normal copy. Finally, we used real-time PCR to quantify the *KIT* copy number variation in cells edited by CRISPR/Cas9. We found all sgRNAs were able to reduce the copy number efficiently in sorted cells, with a 13.30% reduction by sgRNA16–1, a 9.20% reduction by sgRNA16–2, a 12.40% reduction by sgRNA17–6, and a 4.90% reduction by sgRNA17–8 (Fig. 3d). These results indicate the possibility of obtaining cells with corrected structural mutations at the *KIT* locus.

### Generation of single cell clones with corrected KIT structural mutations

**Fig. 4:**
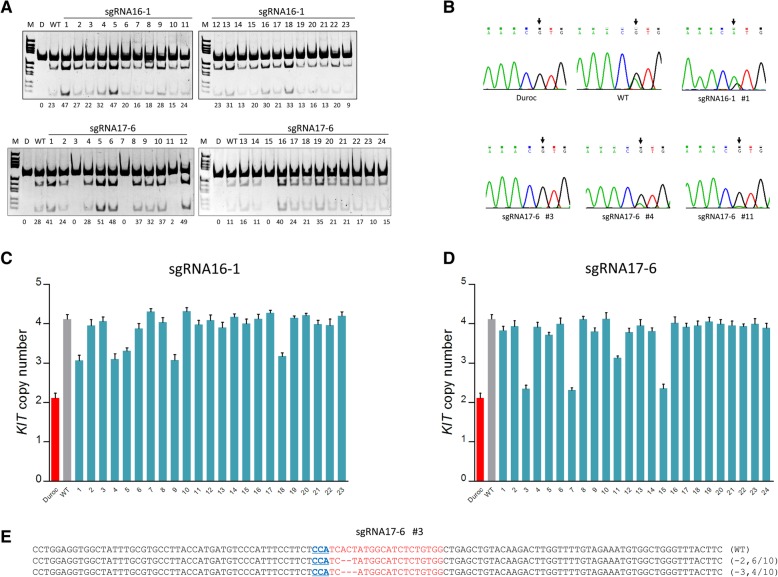
Analysis of *KIT* copy variations in single cell clones. **a** Variation of the percentage of *KIT* copy with the splice mutation single cell clones as determined by the *Nla* III assay. D, duroc (pig breed with wild-type *KIT* allele); WT, non-edited Yorkshire pig kidney cells. **b** Variation of the percentage of the splice mutation in each single cell clone as reflected by the sequencing chromatograms. Black arrows indicate the G > A splice mutation. **c** *KIT* copy number in each single cell clone derived from cells edited by sgRNA16–1 determined by qPCR. (T-test, p < 0.05) (**d**) *KIT* copy number in each single cell clone derived from cells edited by sgRNA17–6 determined by qPCR. (T-test, p < 0.05) (**e**) Sequence analysis of cloned PCR products. DNA sequences of the wild-type (WT) and mutant clones, with CRISPR/Cas9 recognition sites shown in red and PAM sequences in blue. Dashes indicate deleted bases

Since sgRNA16–1 and sgRNA17–6 were found to induce copy number reductions of the *KIT* locus relatively more efficiently, single cell clones were generated from cells transfected with either Cas9/sgRNA16–1 or Cas9/sgRNA17–6 (Additional file 4: Figure. S3). An *Nla* III assay was first applied to detect whether the *KIT* copy with the splice mutation was completely removed from the genome. As expected, none of the 23 single cell clones derived from cells edited by Cas9/sgRNA16–1 were resistant to *Nla* III digestion. In contrast, 12.5% (3/24) of the single cells derived from cells edited by Cas9/sgRNA17–6 presented complete resistance to *Nla* III digestion (Fig. 4a). This result demonstrates that the *KIT* copy with splice mutation is downstream of the normal copy, and that it can be completely removed through large fragment deletion induced by sgRNA targeting intron 17 (Fig. 2b). Sequencing analysis of the splice mutation site in each clone confirmed that the mutated nucleotide A was absent in clones resistant to *Nla* III digestion (e.g. sgRNA17–6 #3 clone); the percentage of mutated nucleotide A decreased in clones with reduced sensitivity to *Nla* III digestion (e.g. sgRNA17–6 #11 clone); and the percentage of mutated nucleotide A increased in clones with increased sensitivity to *Nla* III digestion (e.g. sgRNA16–1 #1 clone) (Fig. 4b). The copy number of the *KIT* locus in each single cell clone was quantified by qPCR (Fig. 4c and d). Consistent with the *Nla* III assay, out of the 24 single cell clones, the copy number in the 3 single cell clones presenting complete resistance to *Nla* III digestion, was corrected back to the normal two. In addition, in one single cell clone edited by Cas9/sgRNA17–6, the copy number was reduced from 4 to 3, consistent with its reduced sensitivity to *Nla* III digestion. Thus, taken together, Cas9/sgRNA17–6 was capable of inducing the deletion of the *KIT* copy with splice mutation at a frequency of 16.7% (4/24). Moreover, Cas9/sgRNA16–1 was capable of removing one duplicated *KIT* copy from the genome at a frequency of 21.7% (5/23). In the single cell clone (sgRNA17–6 #3) with corrected *KIT* structural mutations, in each allele, only small deletions (2 and 3 bases deletions) were found around the cutting site of sgRNA17–6 (Fig. 4e). Small modifications at intron 17 generally do not affect the expression of the *KIT* gene.

### Off-target effect analysis

The off-target effects (OTE) of CRISPR/Cas9 could potentially affect the health of genome-edited animals. We thus analysed the potential off-target sites (OTS) in the sorted cells by analysing each of the five top-scoring loci of sgRNA16–1 and sgRNA17–6. The T7E1 assay results indicated that sgRNA16–1 could induce unintended cleavage at OTS3 and OTS4, while sgRNA17–6 was unable to induce unintended cleavage at any of the five analysed OTS (Additional file 5: Figure S4A). Sequencing analysis demonstrated that sgRNA16–1 could only induce unintended cleavage at OTS4 but not OTS3 (Additional file 5: Figure S4B). In order to further confirm the specificity of sgRNA 17–6, we randomly selected 5 OTS with high, medium, or low scores for the T7E1 assay, and found that none of these OTS were able to induce unintended cleavage (Additional file 6: Figure S5 and Additional file 1: Table S5). Therefore, in order to minimize the potential off-target effect of edited genomes on the health of pigs, sgRNA17–6, but not sgRNA16–1, was used for the establishment of Yorkshire kidney cells with normal *KIT* copies for the future generation of edited pigs.

## Discussion

The domestication and selection of pigs has resulted in a large variety of coat colours and patterns that are characteristic to different pig breeds and populations [12, 15]. Pig coat colour has been the focus of genetics studies for decades, and with the help of molecular genetics, scientists have identified the genes and mutations responsible for most of the coat colours and patterns found in pigs [6]. Structural mutations of the *KIT* gene have been suggested to play major roles in determining the white coat colour in pigs [14]. However, the functional study of these mutations has not yet been carried out, most likely due to difficulty associated with correcting a 450-kb fragment duplication using conventional genetic engineering technology. With the advent of the CRISPR/Cas9 system, a versatile genome-editing tool, scientists are now capable of generating a variety of mutations, including structural mutations, in mammalian genomes. In recent years, CRISPR/Cas9 has been successfully used to generate a 350-kb deletion in the mice *LAF4* gene to obtain Nievergelt Syndrome [18], which is one example of rapid in vivo modelling of genomic rearrangements. The successful deletion of the duplicated 450-kb *KIT* copy in our study confirmed the advantages of CRISPR/Cas9 in the engineering of structural variants.

Chromosome deletion usually relies on the cellular delivery of a pair of sgRNAs to create two DSBs at a locus in order to delete the intervening DNA segment by NHEJ repair [1]. In this study, we used single sgRNA for the deletion of duplicated copies of a large DNA fragment. This is a relatively easier and more efficient method for cell transfection than the transfection of a pair of sgRNAs. We successfully deleted two duplicated copies of the 450-kb *KIT* locus in porcine primary cells at a frequency of 12.5%, which is comparable to previous reports on kilobase-size deletions in other cell types with efficiencies ranging from 1 to 13% [7, 8, 18, 23]. To the best of our knowledge, this is the first report regarding the engineering of structural variations in the genomes of livestock.

## Conclusions

In conclusion, we used CRISPR/Cas9 for the efficient correction of structural mutations in the 450-kb *KIT* locus, providing donor cells for the creation of genome-edited Yorkshire pigs with normal *KIT* copies. This provides a basis for the further investigation of the underlying genetic mechanisms of porcine coat colour and the possibility for the generation of a new breed of Yorkshire pigs with wild-type coat colour.

## Methods

### sgRNA design and vector construction

Guide sequences for two sgRNAs (sgRNA16–1, sgRNA16–2) targeting intron 16 and two sgRNAs (sgRNA17–1, sgRNA17–2) targeting intron 17 of the porcine *KIT* gene were selected using an open tool: CRISPR DESIGN (https://benchling.com/crispr). The oligos of each sgRNA guide sequence were cloned downstream of the human U6 promoter via *Bbs* I restriction sites in the plasmid pSpCas9(BB)-2A-GFP (pX458) (Addgene plasmid #48138) to create the plasmid pX458-sgRNA. Positive clones were confirmed by Sanger sequencing (Sangon, China). sgRNA sequences and details were listed in Additional file 1: Table S1.

### Porcine kidney cell culture, transfection, and sorting

Two New born Yorkshire piglets were purchased from Guangxi yangxiang Technology Co., Ltd. (China). After sacrificing these piglets, porcine kidney cells were isolated from kidneys and cultured in Dulbecco’s modified Eagle medium (Gibco, USA) supplemented with 100 units ml^− 1^ penicillin, 100 μg ml^− 1^ streptomycin (Gibco, USA), and 10% foetal bovine serum (Gibco, USA) at 37 °C under a 5% CO_2_ humidified atmosphere (Thermo, USA). The animal study was supervised the Institutional Animal Care and Use Committee of the Sun Yat-sen University (approval no. IACUC DD-17-0403) and used in accordance with regulation and guidelines of this committee. For electroporation, porcine kidney cells were harvested and counted, and 1 × 10^6^ cells were resuspended in 100 μl buffer R (Invitrogen, USA), containing 10 μg pX458-sgRNA plasmid. The mixture was then transfected through electroporation at 1650 V for 10 ms in 3 pulses using the Neon transfection system (Invitrogen, USA) and seeded into 6-well plates (Nunc, USA) with 2 ml preheated culture medium. After 24 h of transfection, the culture medium was refreshed gently to exclude dead cells. Cells were then observed and photographed with a fluorescence microscope (Nikon, Japan). After 48 h of transfection, cells were dissociated with trypsin (Sigma, USA) at 37 °C for 4 min and resuspended in PBS (Gibco, USA), then analysed and collected by fluorescence-activated cell sorting (FACS) using Aria II cell sorter (BD Biosciences, USA). EGFP-positive cells were sorted into 1.5-ml centrifuge tubes and centrifuged either for further culturing or used for the isolation of genomic DNA. The single cell was seeded into 96-well plates using Aria II cell sorter. After three weeks of culture, the single cell was expanded for subsequent analysis.

### T7E1 assay and Nla III assay

Genomic DNA samples were extracted from EGFP positive cell populations using the DNeasy Blood & Tissue Kit (Qiagen, Germany) according to the manufacturer’s instructions. The targeted sites were amplified by PrimerSTAR HS DNA polymerase (TaKaRa, Japan) with the primer pairs and purified with a gel extraction kit (Omega, USA). Then, 300 ng purified PCR products for T7 endonuclease I (T7E1) assay were denatured and annealed in NEBuffer 2 using a thermocycler (Bio-Rad, USA), then digested with T7E1 (NEB, UK) for 30 min at 37 °C and separated by 10% native polyacrylamide gel electrophoresis (native-PAGE). Mutation frequencies were calculated based on the band intensities using Image J software and then PCR products were cloned into a pMD-18 vector (Takara, Japan) and sequenced to confirm the mutation efficiency by dividing the number of mutant clones by the number of total clones. Primers used for PCR are listed in Additional file 1: Table S2.

The G > A mutation in the first base of intron 17 of *KIT* introduces the restriction site *Nla* III. We amplified a 145 bp fragment across the splice mutation site and digested the PCR products using the *Nla* III enzyme to determine the efficiency of the deletion of *KIT* copies with G > A mutation by CRISPR/Cas9 (Fig. 3a). A complete deletion of *KIT* copies with the G > A mutation would eliminate the restriction site, which is detected as a failure to cleave the PCR product by *Nla* III. In contrast, a complete deletion of a normal *KIT* copy would result in complete digestion of the PCR product by *Nla* III. Purified PCR products for *Nla* III assay were amplified and digested with *Nla* III (Thermo, USA) for 5 min at 37 °C and separated by 15% native-PAGE. The primers used for PCR are listed in Additional file 1: Table S3.

### Real-time quantitative PCR (qPCR) analysis

Copy number variation was estimated using real-time quantitative PCR and the 2^-△△CT^ method as described by Livak and Soejima [13, 22]. The primers were designed using Primer-BLAST on NCBI and the primer details for *KIT* (Genbank accession number: CU929000.2) and *COL10A1* (Genbank accession number: AF222861.1) are listed in (Additional file 1: Table S4). The copy number of c-kit was normalized against the Col10 region, a control region in the genome that did not vary in copy number between the pigs [4]. The PCR reaction was performed using the Roch LC480 in 20 μl reaction volumes using ChamQ™ SYBR qPCR Master Mix (Vazyme, China). The procedure in the thermal cycling was an initial 5 min hold at 95 °C, followed by 40 cycles of 15 s at 95 °C, 30 s at 60 °C, and 30 s at 72 °C.

### Off-target assay

To determine the site-specific cleavage of the CRISPR/Cas9 system in vitro, potential off-target sites (Additional file 1: Table S5) were evaluated by CRISPR DESIGN (https://benchling.com/crispr). Each five top-scoring off-target sites of sgRNA16–1 or sgRNA17–6 were selected for the T7E1 assay (Additional file 1: Table S6) and those yielding typical cleavage bands were considered as candidates. Finally, the PCR products of the candidates were sequenced to confirm the off-target effects. Further confirmation of the targeting specificity of sgRNA17–6 was carried out by analysing each five off-target sites with high, medium, or low scores (Additional file 1: Table S5) by T7E1 assay (Additional file 1: Table S6).

## Additional files

Additional file 1: **Table S1.** List of sgRNAs designed for targeting the intron16 and intron17 of *KIT* gene. **Table S2.** Primers used for T7E1 assay. **Table S3.** Primers used for *Nla* III assay. **Table S4.** Primers used for qPCR. **Table S5.** List of the potential off-target sites. **Table S6.** Primers used for off-target effects assay. (DOCX 27 kb)

Additional file 2: **Figure S1.** Fluorescent images of porcine kidney cells 24 h after transfection of plasmid pX458-sgRNAs. (JPG 2434 kb)

Additional file 3: **Figure S2.** Flow cytometry of porcine kidney cells 48 h after transfection of plasmid pX458-sgRNAs. The percentage of cells expressing EGFP is noted. (JPG 2023 kb)

Additional file 4: **Figure S3.** Images of cell clones expanded from single porcine kidney cell. One week’s culture of single cell seeded each well of 96-well plates through FACS. (JPG 3173 kb)

Additional file 5: **Figure S4.** Detection of the potential off-target effects of sgRNA16–1 and sgRNA17–6. (A) T7E1 assay for the analysis of potential off-target effects. NC indicates the negative controls. Untransfected cells were used as negative controls. OTS1, OTS2, OTS3, OTS4, and OTS5 indicate the experimental groups transfected with each pX458-sgRNAs. M, DNA marker. Red arrowheads indicate the expected cleaved bands by T7E1. (B) Sequencing analysis of the potential mutations on OTS3 and OTS4 induced by sgRNA16–1. Black lines indicate the potential binding sequences of sgRNA16–1 on OTS3 and OTS4, and red lines indicate PAM sequences. Yellow arrowheads indicate the sgRNA cutting sites. In the sequencing chromatograms, double peaks at cutting sites indicate indels induced at the cutting site. (JPG 691 kb)

Additional file 6: **Figure S5.** Further detection of the potential off-target effects of sgRNA17–6. NC indicates the negative controls. Untransfected cells were used as negative controls. OTS indicates the experimental groups transfected with each pX458-sgRNAs. M, DNA marker. Red arrowheads indicate the expected cleaved bands by T7E1. (JPG 1060 kb)
