# Supplementary material for: Highly efficient correction of structural mutations of 450 kb KIT locus in kidney cells of Yorkshire pig by CRISPR/Cas9
Source: BMC Mol Cell Biol. 2019 Apr 3;20:4. doi: 10.1186/s12860-019-0184-5 (PMC6446502; doi:10.1186/s12860-019-0184-5)
Supplement: Supplementary file 1 — Table S1. List of sgRNAs designed for targeting the intron16 and intron17 of KIT gene. Table S2. Primers used for T7E1 assay. Table S3. Primers used for Nla III assay. Table S4. Primers used for qPCR. Table S5. List of the potential off-target sites. Table S6. Primers used for off-target effects assay. (DOCX 27 kb) [file 12860_2019_184_MOESM1_ESM.docx]

**Supplementary Tables**

**Table S1.** List of sgRNAs designed for targeting the intron16 and intron17 of *KIT* gene.

| Name | Target sequence (5´-3´) | Strand | GC content (%) | PAM |
| --- | --- | --- | --- | --- |
| sgRNA16-1 | AGTGGAGGTGATTCTCATGG | + | 50% | AGG |
| sgRNA16-2 | GGCTCTAAAATGCTCCTTGG | - | 50% | GGG |
| sgRNA17-6 | CCACAGAGATGCCATAGTGA | - | 50% | TGG |
| sgRNA17-8 | GCCTTTGAACATCCACAAAG | + | 45% | GGG |

**Table S2**. Primers used for T7E1 assay.

| Name | Sequence (5´-3´) | Size (bp) | AT(°C) |
| --- | --- | --- | --- |
| *KIT*16-F1 | CAGGCTCATACATAGAACGAGATG | 511 | 60 |
| *KIT*16-R1 | CAGGCACAGGCTTCACTGA |  |  |
| *KIT*17-F3 | TTAGTGATGGCTGTCTGAGATGA | 580 | 60 |
| *KIT*17-R3 | AGGAGGCAGGTGACCGTATTATTAC |  |  |

**Table S3.** Primers used for *Nla* III assay

| Name | Sequence (5´-3´) | Size (bp) | AT (°C) |
| --- | --- | --- | --- |
| *Nla* III-F | TAGCCAGAGACATCAAGAAT | 145 | 56 |
| *Nla* III-R | AGGTAGGGTGTGCATTATG |  |  |

**Table S4.** Primers used for qPCR.

| Name | Sequence (5´-3´) | Size (bp) | AT(°C) |
| --- | --- | --- | --- |
| q*KIT*-F | GTCAGTGCTGGCGATGAGATTAG | 75 | 60 |
| q*KIT*-R | ACCCAGGGTCTCAAAAGTCCAT |  |  |
| q*COL*-F | AAGCTTCAAACAGGGGTACAAT | 84 | 60 |
| q*COL*-R | CCACTTGGAATGTTACCCTAATG |  |  |

**Table S5.** List of the potential off-target sites

| 16-1 | Sequence (5´-3´) | Score | Mismatches | UCSC gene |
| --- | --- | --- | --- | --- |
| OTS1 | AATTCAGTTGATTCTCATGGTGG | 1.4 | 4MMs [2:4:5:8] | / |
| OTS2 | TGCATAGGTGATTCTCATGGGAG | 1.3 | 4MMs [1:3:4:5] | / |
| OTS3 | CGTGGGGGTGATTCTCATGCAGG | 0.9 | 3MMs [1:6:20] | / |
| OTS4 | AGAGGAAGTGACTCTCATGGGGG | 0.9 | 3MMs [3:7:12] | / |
| OTS5 | GGAGGGGCTGATTCTCATGGAAG | 0.8 | 4MMs [1:3:6:8] | / |
| 17-6 | Sequence (5´-3´) | Score | Mismatches | UCSC gene |
| OTS1 | CCACACTGATGCCATAGTGATGG | 2.2 | 2MMs [6:7] | / |
| OTS2 | TCATAAAGATGCCATAGTGATAG | 1.5 | 3MMs [1:4:6] | / |
| OTS3 | ACACAGAAATGACATAGTGAAGG | 1.4 | 3MMs [1:8:12] | / |
| OTS4 | CTACAGAGAAGCCATAGTGGAAG | 1.4 | 3MMs [2:10:20] | / |
| OTS5 | CATCTGTGATGCCATAGTGATAG | 0.9 | 4MMs [2:3:5:7] | / |
| OTSH1 | CCACACTGATGCCATAGTGATGG  CAGGTGCAGAGATGCAGGAA  CAGGTGCAGAGATGCAGGAA | 2.2 | 2MMs [6:7] | / |
| OTSH2 | ACACAGAAATGACATAGTGAAGG | 1.4 | 3MMs [1:8:12] | / |
| OTSH3 | CCAGGGAGATGTCATAGTGAGGG | 1.4 | 3MMs [4:5:12] | / |
| OTSH4 | CCACTGGGCTGCCATAGTGAAGG | 1 | 3MMs [5:7:9] | / |
| OTSH5 | GACCAGAGCTGCCATAGTGATGG | 0.9 | 4MMs [1:2:3:9] | / |
| OTSM1 | AGAGAGAGATGCCATAATGAGGG | 0.7 | 4MMs [1:2:4:17] | / |
| OTSM2 | CCTCAGTAATCCCATAGTGATGG | 0.6 | 4MMs [3:7:8:11] | / |
| OTSM3 | CAACACAACTGCCATAGTGATGG | 0.6 | 4MMs [2:6:8:9] | / |
| OTSM4 | GCACAGAGATGCCATAATTAAGG | 0.5 | 3MMs [1:17:19] | / |
| OTSM5 | CCAGAGGGAAGCCATAGTGTAGG | 0.5 | 4MMs [4:7:10:20] | / |
| OTSL1 | GCTCAGAAATGCCAGAGTGAGGG | 0.5 | 4MMs [1:3:8:15] | / |
| OTSL2 | CCAAAAAAATGCCATAGTGTTGG | 0.5 | 4MMs [4:6:8:20] | / |
| OTSL3 | CCAAACAGAAGCCATAGTGGAGG | 0.4 | 4MMs [4:6:10:20] | / |
| OTSL4 | TCACTGCGATGCCATAGTAATGG | 0.4 | 4MMs [1:5:7:19] | / |
| OTSL5 | GCATAGAGATGCCATATTGTTGG | 0.4 | 4MMs [1:4:17:20] | / |

**Table S6.** Primers used for off-target effects assay

| Name | Sequence (5-3) | Size(bp) | AT(°C) |
| --- | --- | --- | --- |
| 16-OTS1-F  -F1 | AGCTTGAATGCAGCCTGTCG | 415 | 60 |
| 16-OTS1-R | GGGTAGAAAGGCCTCAGTGTG |  |  |
| 16-OTS2-F | GTGCCTCACTCCTGGTCT | 522 | 60 |
| 16-OTS2-R | CTGCTTTCGCAATCAACT |  |  |
| 16-OTS3-F | TGTGTCGGTGACAAACCCTT | 476 | 60 |
| 16-OTS3-R | GCGCACAGAGGTATAGAGCC |  |  |
| 16-OTS4-F | CTGGGTCTGGATAAGGAGC | 548 | 60 |
| 16-OTS4-R | TCAGCCTGTGGACTTACGG |  |  |
| 16-OTS5-F | ATGTACTGCCTCTGGAAAG | 541 | 60 |
| 16-OTS5-R | CTTGGGATGGAGCATGATGGAAG |  |  |
| 17-OTS1-F | TTGAGAACCACTGCCTTGC  TTGAGAACCACTGCCTTGC | 464 | 60 |
| 17-OTS1-R | TTCCCTCTTATCCCATCCC |  |  |
| 17-OTS2-F | AGATAATTTGGTGAGGGTC | 394 | 60 |
| 17-OTS2-R | TTGGGTTTCTTCAAGGAGTT |  |  |
| 17-OTS3-F | CACTGTCAGTTTTGAGGGGAAA | 572 | 60 |
| 17-OTS3-R | AATCAAAGCATCAAAGAGCCAC |  |  |
| 17-OTS4-F | GGCAGCATCCCATCTCATA | 535 | 60 |
| 17-OTS4-R | AAGGACGGAGCAGAATCAA |  |  |
| 17-OTS5-F | AGATACAGTGAAACCCAAAG | 477 | 60 |
| 17-OTS5-R | TGGAGGACTGGCTGGAGGT |  |  |
| 17-OTSH1-F | CAGGTGCAGAGATGCAGGAA | 700 | 60 |
| 17-OTSH1-R | CCGTCAGTCCTCTCCTTTGG |  |  |
| 17-OTSH2-F | TGGACTCCGACATAGCCCTT | 941 | 60 |
| 17-OTSH2-R | ATCGTGAATCCAGCCGTTCA |  |  |
| 17-OTSH3-F | TTAGTAGGCCAGCCCAGGAT | 830 | 60 |
| 17-OTSH3-R | TTGTGCACTGGTGTTCCCTT |  |  |
| 17-OTSH4-F | GCTCCTCACCTGCTTTCCAA | 604 | 60 |
| 17-OTSH4-R | CTGGCAATACCTGCCTCAGT |  |  |
| 17-OTSH5-F | ACTCACTGTGTTGCCAGAGG | 774 | 60 |
| 17-OTSH5-R | AACCTGATGGTCACCTGCAC |  |  |
| 17-OTSM1-F | CATGCATCCCGTTCACTCAC | 500 | 60 |
| 17-OTSM1-R | TGTCACCCCTTGAGACAGTG |  |  |
| 17-OTSM2-F | GCCAGAGCGCATTCTCATTG | 327 | 60 |
| 17-OTSM2-R | GAACTGAAAACTCGCTGCCC |  |  |
| 17-OTSM3-F | GCATCCGTGTTCTCATCCCA | 548 | 60 |
| 17-OTSM3-R | AAGCTGAAAGTAGACGGGGC |  |  |
| 17-OTSM4-F | AAGGTGTCCCCCTCTTTGGG | 687 | 60 |
| 17-OTSM4-R | CCAGAAGGCATCAAGCACCTAT |  |  |
| 17-OTSM5-F | CCGAGGGTCAAATTAAACACA | 804 | 60 |
| 17-OTSM5-R | AGTGCATTCTTTTAAACCCAA |  |  |
| 17-OTSL1-F | AGCAGTCCCACCTGAATCTG | 736 | 60 |
| 17-OTSL1-R | CTCAGCAATCTACGAGGTGATA |  |  |
| 17-OTSL2-F | GACGTGCAGGCTGTATTTAGA | 552 | 60 |
| 17-OTSL2-R | CACCAGAGCCCATCATGACTA |  |  |
| 17-OTSL3-F | TCTGAAAAACCAAGAGACCGCT | 402 | 60 |
| 17-OTSL3-R | CCAGCTTCCTCTTGAGGCTTAAT |  |  |
| 17-OTSL4-F | TATGCCACCGTCTGTTTGCT | 499 | 60 |
| 17-OTSL4-R | ACTCTCCTTGGGTCCCTGAA |  |  |
| 17-OTSL5-F | GCAACGAGTTCCATGTCAGC | 732 | 60 |
| 17-OTSL5-R | CAAGCCAAACACAACCCACC |  |  |
