# Supplementary figures and images for: Highly efficient correction of structural mutations of 450 kb KIT locus in kidney cells of Yorkshire pig by CRISPR/Cas9
Source: BMC Mol Cell Biol. 2019 Apr 3;20:4. doi: 10.1186/s12860-019-0184-5 (PMC6446502; doi:10.1186/s12860-019-0184-5)

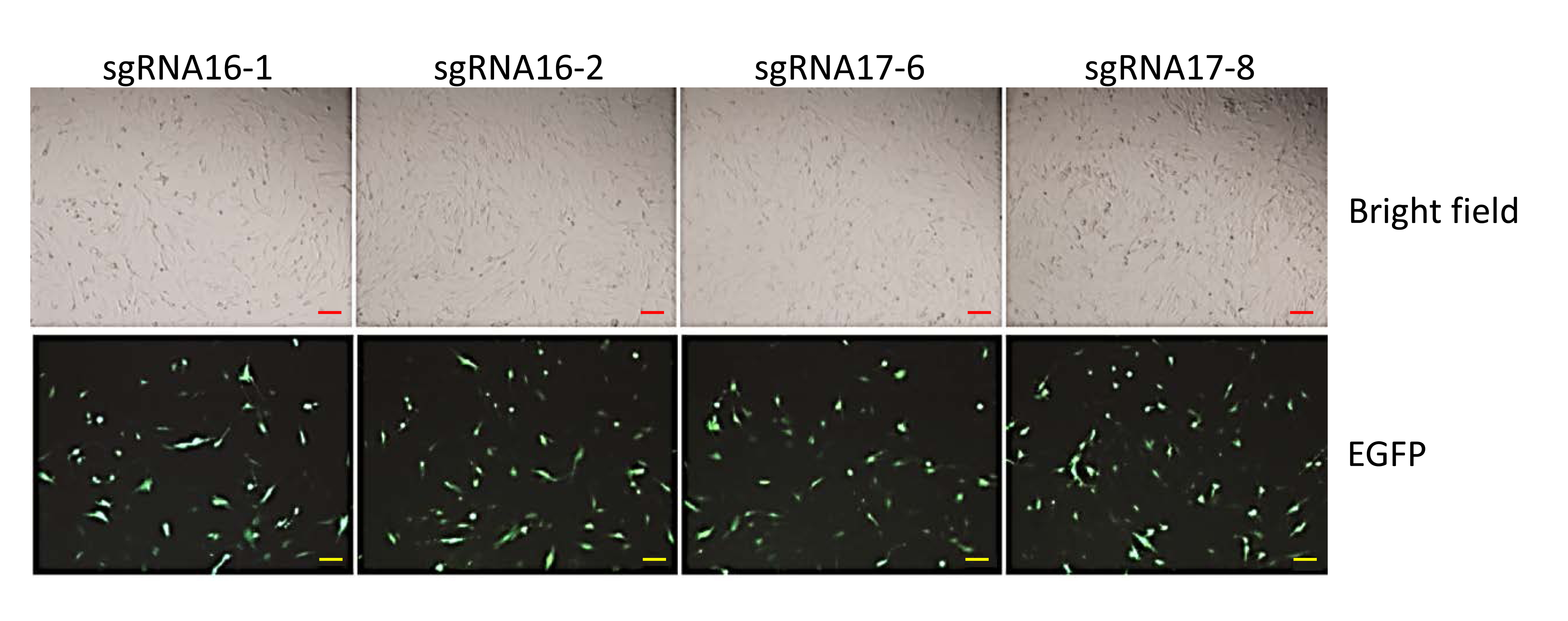

Supplement: Supplementary file 2 — Figure S1. Fluorescent images of porcine kidney cells 24 h after transfection of plasmid pX458-sgRNAs. (JPG 2434 kb) [file 12860_2019_184_MOESM2_ESM.jpg]

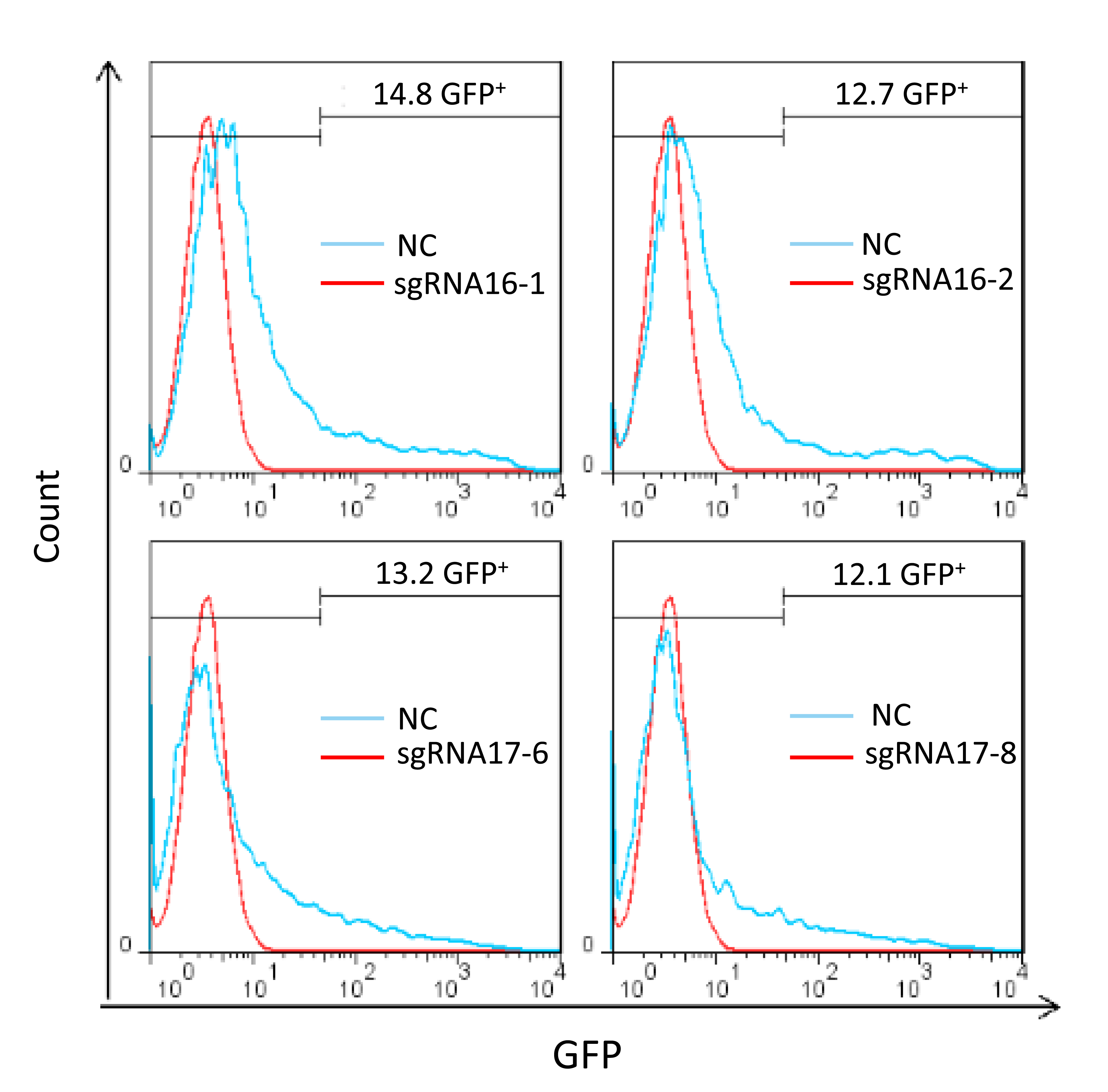

Supplement: Supplementary file 3 — Figure S2. Flow cytometry of porcine kidney cells 48 h after transfection of plasmid pX458-sgRNAs. The percentage of cells expressing EGFP is noted. (JPG 2023 kb) [file 12860_2019_184_MOESM3_ESM.jpg]

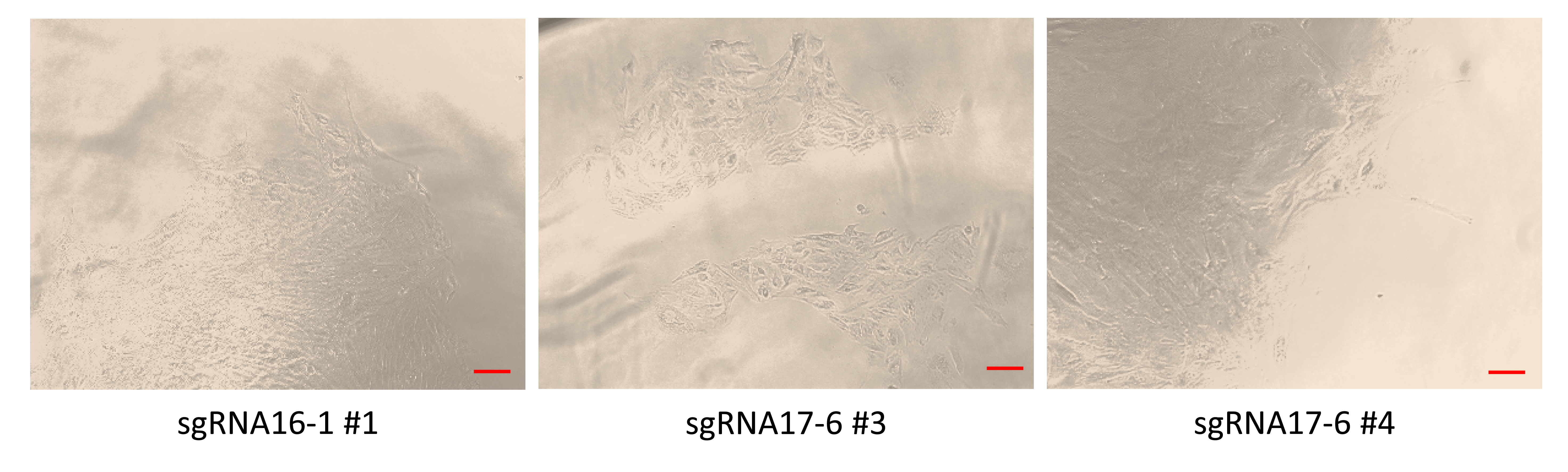

Supplement: Supplementary file 4 — Figure S3. Images of cell clones expanded from single porcine kidney cell. One week’s culture of single cell seeded each well of 96-well plates through FACS. (JPG 3173 kb) [file 12860_2019_184_MOESM4_ESM.jpg]

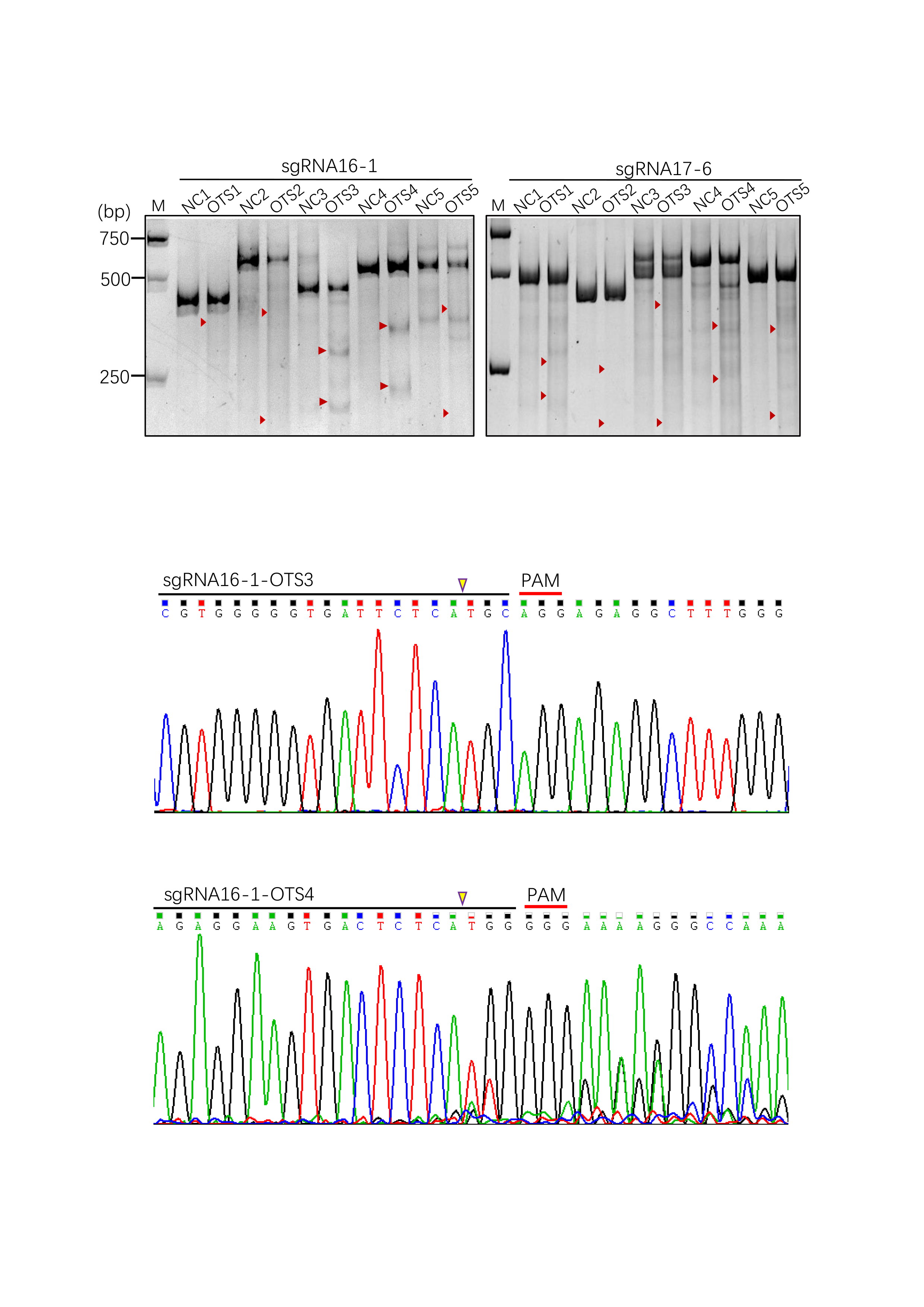

Supplement: Supplementary file 5 — Figure S4. Detection of the potential off-target effects of sgRNA16–1 and sgRNA17–6. (A) T7E1 assay for the analysis of potential off-target effects. NC indicates the negative controls. Untransfected cells were used as negative controls. OTS1, OTS2, OTS3, OTS4, and OTS5 indicate the experimental groups transfected with each pX458-sgRNAs. M, DNA marker. Red arrowheads indicate the expected cleaved bands by T7E1. (B) Sequencing analysis of the potential mutations on OTS3 and OTS4 induced by sgRNA16–1. Black lines indicate the potential binding sequences of sgRNA16–1 on OTS3 and OTS4, and red lines indicate PAM sequences. Yellow arrowheads indicate the sgRNA cutting sites. In the sequencing chromatograms, double peaks at cutting sites indicate indels induced at the cutting site. (JPG 691 kb) [file 12860_2019_184_MOESM5_ESM.jpg]

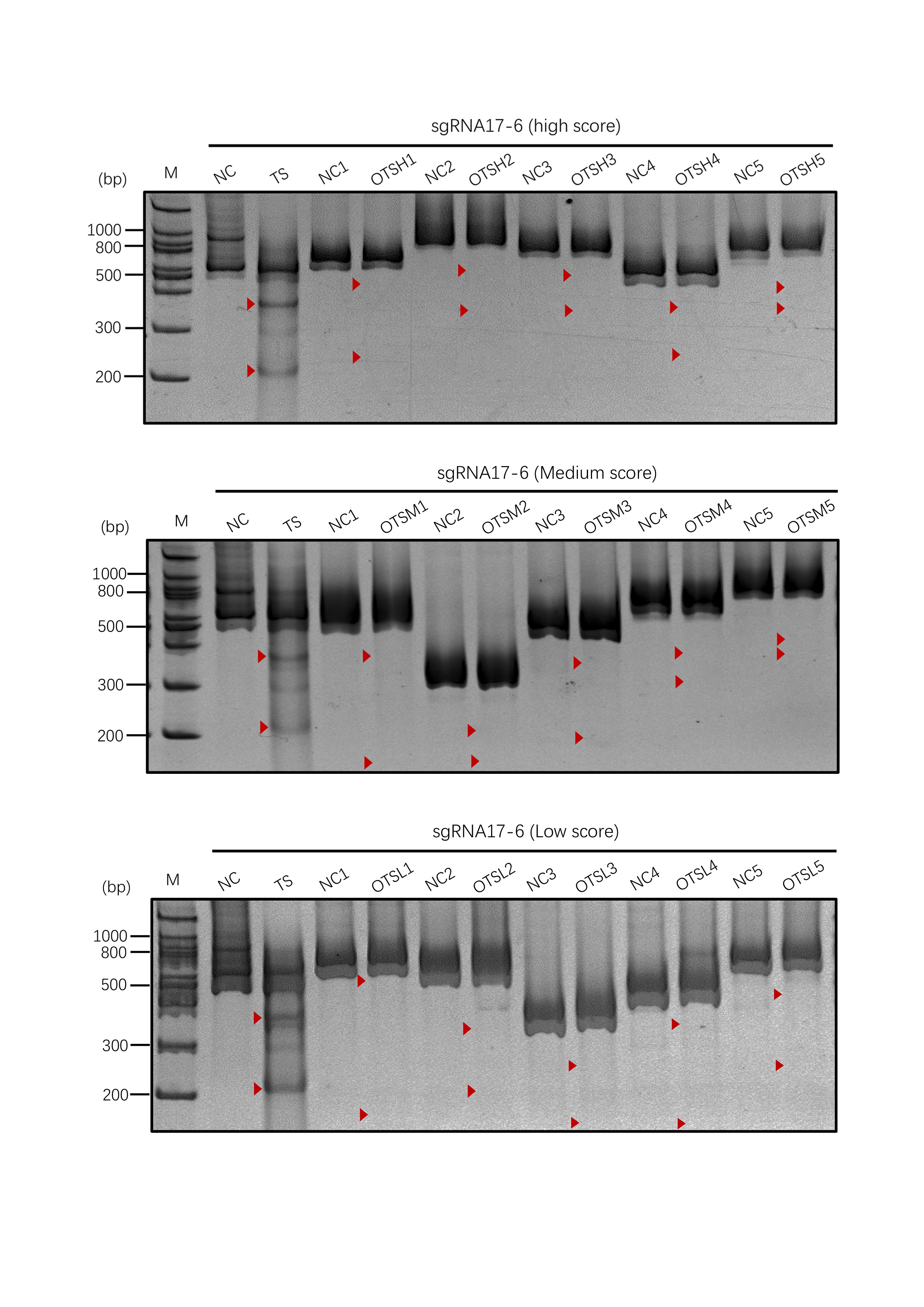

Supplement: Supplementary file 6 — Figure S5. Further detection of the potential off-target effects of sgRNA17–6. NC indicates the negative controls. Untransfected cells were used as negative controls. OTS indicates the experimental groups transfected with each pX458-sgRNAs. M, DNA marker. Red arrowheads indicate the expected cleaved bands by T7E1. (JPG 1060 kb) [file 12860_2019_184_MOESM6_ESM.jpg]
